# Return to work after Post-COVID: describing affected employees' perceptions of personal resources, organizational offerings and care pathways

**DOI:** 10.3389/fpubh.2023.1282507

**Published:** 2023-11-27

**Authors:** Claudia Straßburger, Daniel Hieber, Maximilian Karthan, Markus Jüster, Johannes Schobel

**Affiliations:** ^1^Department of Tourism Management, Kempten University of Applied Sciences, Kempten, Germany; ^2^DigiHealth Institute, Neu-Ulm University of Applied Sciences, Neu-Ulm, Germany; ^3^Institute of Clinical Epidemiology and Biometry, University of Würzburg, Würzburg, Germany; ^4^Department of Pathology, Medical Faculty, University of Augsburg, Augsburg, Germany

**Keywords:** return to work, work ability, post-COVID syndrome, long COVID, occupational health

## Abstract

**Background:**

Most individuals recover from the acute phase of infection with the SARS-CoV-2 virus, however, some encounter prolonged effects, referred to as the Post-COVID syndrome. Evidence exists that such persistent symptoms can significantly impact patients' ability to return to work. This paper gives a comprehensive overview of different care pathways and resources, both personal and external, that aim to support Post-COVID patients during their work-life reintegration process. By describing the current situation of Post-COVID patients pertaining their transition back to the workplace, this paper provides valuable insights into their needs.

**Methods:**

A quantitative research design was applied using an online questionnaire as an instrument. Participants were recruited via Post-COVID outpatients, rehab facilities, general practitioners, support groups, and other healthcare facilities.

**Results:**

The analyses of 184 data sets of Post-COVID affected produced three key findings: (1) The evaluation of different types of personal resources that may lead to a successful return to work found that particularly the individuals' ability to cope with their situation (measured with the FERUS questionnaire), produced significant differences between participants that had returned to work and those that had not been able to return so far (F = 4.913, *p* = 0.001). (2) In terms of organizational provisions to facilitate successful reintegration into work-life, predominantly structural changes (i.e., modification of the workplace, working hours, and task) were rated as helpful or very helpful on average (mean_*workplace*_ 2.55/SD = 0.83, mean_*working hours*_ 2.44/SD = 0.80; mean_*tasks*_ 2.55/SD = 0.83), while the remaining offerings (i.e., job coaching or health courses) were rated as less helpful or not helpful at all. (3) No significant correlation was found between different care pathways and a successful return to work.

**Conclusion:**

The results of the in-depth descriptive analysis allows to suggests that the level of ability to cope with the Post-COVID syndrome and its associated complaints, as well as the structural adaptation of the workplace to meet the needs and demands of patients better, might be important determinants of a successful return. While the latter might be addressed by employers directly, it might be helpful to integrate training on coping behavior early in care pathways and treatment plans for Post-COVID patients to strengthen their coping abilities aiming to support their successful return to work at an early stage.

## 1 Introduction

The global impact of the COVID-19 pandemic has been unprecedented, affecting millions of individuals and profoundly reshaping societal as well as occupational norms (1). While much attention has been focused on preventing and treating acute cases of COVID-19, a significant number of individuals who have recovered from the initial infection continue to experience persistent symptoms and functional limitations (2). These lingering effects, commonly referred to as Post-COVID syndrome or long COVID (3, 4), have emerged as a significant health concern with implications for individuals' ability to resume their normal daily activities, including returning to work (5).

Emerging evidence indicates that 5–10 percent of patients suffer from the so-called Post-COVID syndrome, i.e., experience persistent symptoms for more than 3 months after the infection with the SARS-CoV-2 virus (4, 6). Post-COVID syndrome may manifest in a broad range of symptoms, such as shortness of breath, post-exercise malaise, cognitive decline, chronic fatigue, musculoskeletal pain, and mental health deterioration (7). These symptoms can vary in severity and duration, creating unique challenges for individuals seeking to resume their professional roles (8). Physical limitations and reduced stamina may hinder their ability to perform previously manageable tasks. Cognitive impairments, such as difficulties with concentration and memory, can impact job performance and decision-making abilities. Furthermore, the emotional toll of the illness, combined with the uncertainties surrounding long-term health outcomes, may contribute to increased anxiety, stress, and reduced confidence among affected individuals (9). Previous studies on the Post-COVID syndrome address different realms. First, a lot of research deals primarily with the treatment of Post-COVID. Those studies predominately consider the medical symptoms (i.e., coughs, embolisms, coronary artery diseases) (3, 4), cluster symptoms and cohorts (7, 10, 11), and focus on developing treatment guidelines (e.g., the German S1 guideline or the UK NICE recommendations). Other studies analyze psychological factors after a COVID infection, especially after long-term treatments (i.e., anxiety) (12, 13). Another research field explores determinates or predictors for developing the Post-COVID syndrome. Here, previous studies reported disease severity during the acute phase of COVID-19 as one of the strongest predictors of Post-COVID (14–16). In another study, Dias et al. (17) found that hypertension, higher body mass index, lower hemoglobin, female sex, admission to intensive care unit, and longer stay were independent predictors of long COVID. Other research focuses on the effects of the Post-COVID syndrome and its different outcomes. Here, studies have assessed the patients' quality of life and found that inferior quality of sleep (18), pain and discomfort (19), or chronic exhaustion (20) are primary reasons for diminished quality of life. Eventually, limited research exists assessing occupational cohorts affected by the Post-COVID syndrome and the effects on their work ability and issues of returning to work after or with the Post-COVID syndrome. Here, Gualano et al. (21) provided with their systematic review of existing literature a comprehensive overview highlighting that Post-COVID is a rising problem in occupational medicine, with consequences on workers' quality of life but also on productivity. In this context, Tabacof et al. (22) found that the Post-COVID syndrome negatively impacts physical function, cognitive function, health-related quality of life, and also participation, which are all determinates that eventually influence workers' productivity. In a case study of a long COVID patient returning to work, Tan and Koh (23) described the challenges and occupational health issues that occurred on his way back to corporate life. The authors found that managing the return to work of Post-COVID affected employees is a highly individual task and requires a multidisciplinary approach.

Nevertheless, there is a lack of occupational reintergation programs particularly for Post-COVID affected employees that draw upon multidisciplinary research from fields such as medicine, psychology, occupational health, and rehabilitation. Public health researchers largely attribute this to the neglect of participatory research that focuses on the patients' perspective and voice and identifies their personal and external resources that might restore their work ability (24). However, understanding the strategies employed by Post-COVID patients as well as their subjective views on the effectiveness of different care pathways and organizational offerings can inform the development of such evidence-based programs. Therefore, the research aims of this study were to

(1) describe personal resources and stressors that might facilitate or hinder the Post-COVID patients' return to work,
(2) identify external support programs that can aid individuals in navigating their way back to work,
(3) feature the patients' different (medical) care pathways and subjective ratings of these offerings pertaining to their return to work.

By doing so, this scientific paper contributes to the collective knowledge base surrounding the return-to-work process. It aims to inspire future research, encourage collaboration among various stakeholders, and inform the development of evidence-based programs and policies that enhance the work experiences and well-being of individuals recovering from the Post-COVID syndrome. Eventually, it seeks to raise awareness among employers, healthcare providers, and policymakers about the specific needs of this population, fostering a proactive approach.

## 2 Materials and methods

### 2.1 Study design and instruments

A cross-sectional quantitative research design was applied using an online questionnaire to describe the situation pertaining the patients' reintegration into work-life. The survey consisted of 148 items clustered in nine scales.

#### 2.1.1 Socio-demographic data

Basic socio-demographics (age, sex, material status, children, education, residency) and information on the participant's current employment situation were collected.

#### 2.1.2 COVID-19 infection

Questions on the initial COVID-19 infection date, information on inpatient healthcare, and the prevalence of a Post-COVID diagnosis were asked. The Gießener Beschwerdefragebogen GBB24 (25) was also applied to evaluate physical health complaints that persisted even 3 months after the initial infection with the virus. The GBB24 comprises a list of 24 complaints. The level of complaints are measured via 5-point likert scale with the following response options 0(“not at all”), 1(“slightly”), 2(“somewhat”), 3(“considerably”), and 4(“very much”).

#### 2.1.3 Care pathways

To enable the comparison of different care pathways, participants were asked to indicate the types of care and support they had received so far and rate how helpful they perceived these measures. In addition to a list of pre-defined options (Post-COVID ambulance, inpatient rehab facility, outpatient rehab facility, support groups, consultation with general practitioner, information via social media, and platforms), participants could also add own items. Finally, they were asked which types of care they would have preferred but had not received.

#### 2.1.4 Health-related quality of life

The SF-12 questionnaire (26) was used to assess physical and mental health functioning and wellbeing to measure the current level of health-related quality of life. The SF-12 is a self-reported outcome measure assessing the impact of health on an individual's everyday life. It comprises of eight domains which are: (1) limitations in physical activities because of health problems, (2) limitations in social activities because of physical or emotional problems, (3) limitations in usual role activities because of physical health problems, (4) bodily pain, (5) general mental health, (6) limitations in usual role activities because of emotional problems, (7) vitality, (8) general health perceptions. The SF-12 is designed as a general measure of health so can be used with the general population.

#### 2.1.5 Stressors

To identify chronic stressors that might impede return to work or arise while returning to work, the Trier Inventory for Chronic Stress (TICS) questionnaire (27) was applied. The TICS is a standardized German questionnaire that has been tested with respect to its factorial structure and psychometric properties, showing good to very good reliability. Internal consistency (Cronbach's Alpha, α) was good to very good with values ranging from 0.84 to 0.91 (mean of α=0.87). Nine interrelated factors of chronic stress are assessed. The nine factors were derived from 57 items rated on a five-point rating scale (1–5, labeled as: “never,” “rarely,” “sometimes,” “frequently,” and “always”). Participants rate the occurrence or frequency of specific situations with a recall period of the previous 3 months. For the present study, four sub-scales (social overload, lack of social recognition, social tension, and social isolation) were selected to control the effect of perceived chronic stress. The remaining 5 scales of TICS handling work-related stress were deliberately excluded, as it was assumed that most participants had been incapable of working for an extended period.

#### 2.1.6 Overall quality of life

The World Health Organization Quality of Life-BREF inventory (WHOQOL-BREF) (28) is a shorter version of the WHOQOL-100. The questions stem from multiple statements about quality of life, health and well-being from people with and without disease, and health professionals. It can be applied for specific populations or groups with a particular disease. The WHOQOL-BREF comprises 26 questions on the individual's perceptions of their health and well-being over the previous two weeks. Responses to questions are on a 1–5 Likert scale where 1 represents “disagree” or “not at all” and 5 represents “completely agree” or “extremely.” In the present study it was applied to measure the quality of life in the five domains. Besides a total quality of life score, sub-scores in physical health, psychological wellbeing, social relationships, and environment were assessed.

#### 2.1.7 Personal resources

The German FERUS questionnaire (Fragebogen zur Erfassung von Ressourcen und Selbstmanagementfähigkeiten) (29) was applied to reveal the participants' health-related resources and self-management skills. The FERUS is a German questionnaire to assess individual resources, like social support and motivation to change, as well as skills in self-management, like coping, introspection, self-efficacy, self-verbalization, and hope. For the current study, only the coping as well as social support scale of the FERUS was used and thus consisted of 22 statements. The degree of consent to each statement is rated on a five-point Likert scale from 1 (“not true”) to 5 (“very true”). Two sub sum-scores were calculated and compared to norm values.

#### 2.1.8 Work ability

The full version of the Work Ability Index WAI (30) was used to assess the participants' current level of work ability. The WAI is an established instrument that underlies the assumption that work ability is represented by the factors of subjective work ability and resources, as well as the health conditions. The WAI consists of 7 items, including current work ability compared with the lifetime best, work ability in relation to the demands of the job, number of current disease groups diagnosed by a physician, estimated work impairment due to diseases, sick leave during the past year, personal prognosis of work ability for 2 years from now and mental resources, referring to the participant's life in general, both at work and during leisure time. The total WAI score is calculated by summing up the scores of all items and is ranged from 7 to 49. The total WAI scores are categorized into 4 levels: poor (7–27), moderate (28–36), good (37–43), and excellent (44–49).

#### 2.1.9 Return to work

In this section, participants were asked to indicate if and to which extent (i.e., full-time, with reduced working hours) they had already returned to work as well as how long it took them to return to work after the initial infection. Additionally, participants were asked how many days of sick leave they had called in after returning to work. Then, the participants had to provide information on organizational offerings (i.e., job coaching, adjustments of working hours, working place or tasks, health consulting, and reintegration plan) they had received to foster a successful return to work. Eventually, they had to rate if these offerings helped them to reintegrate successfully. Finally, they were asked about obstacles they experienced while returning to their workplace.

### 2.2 Participants and procedure

The link to the online questionnaire was distributed through Post-COVID outpatients and inpatient rehab facilities, general practitioners, support groups, and other healthcare facilities to generate a heterogeneous sample. Flyers and posters were handed out to inform potential candidates about the study. Primarily, the German-speaking area (with a strong focus on Germany) was addressed. Inclusion criteria for the study were as follows: (a) participants had to be at least 18 years old, (b) had a confirmed infection with COVID-19 at least three months prior to study enrollment, (c) had or still have self-reported symptoms consistent with Post-COVID syndrome, and (d) have been employed for the last 12 months (even if currently on sick leave).

First, a pre-test was conducted (*n* = 6) to minimize comprehension problems, control for motivational confounding, and assess technical consistency. The study was carried out anonymously. As no personal data was collected, the survey had to be completed in one sitting, with no option to continue later. Per GDPA (Art. 7 §3), participants could drop out at any point in the study. Thereby, no information was persisted on the server.

**Figure 1:**
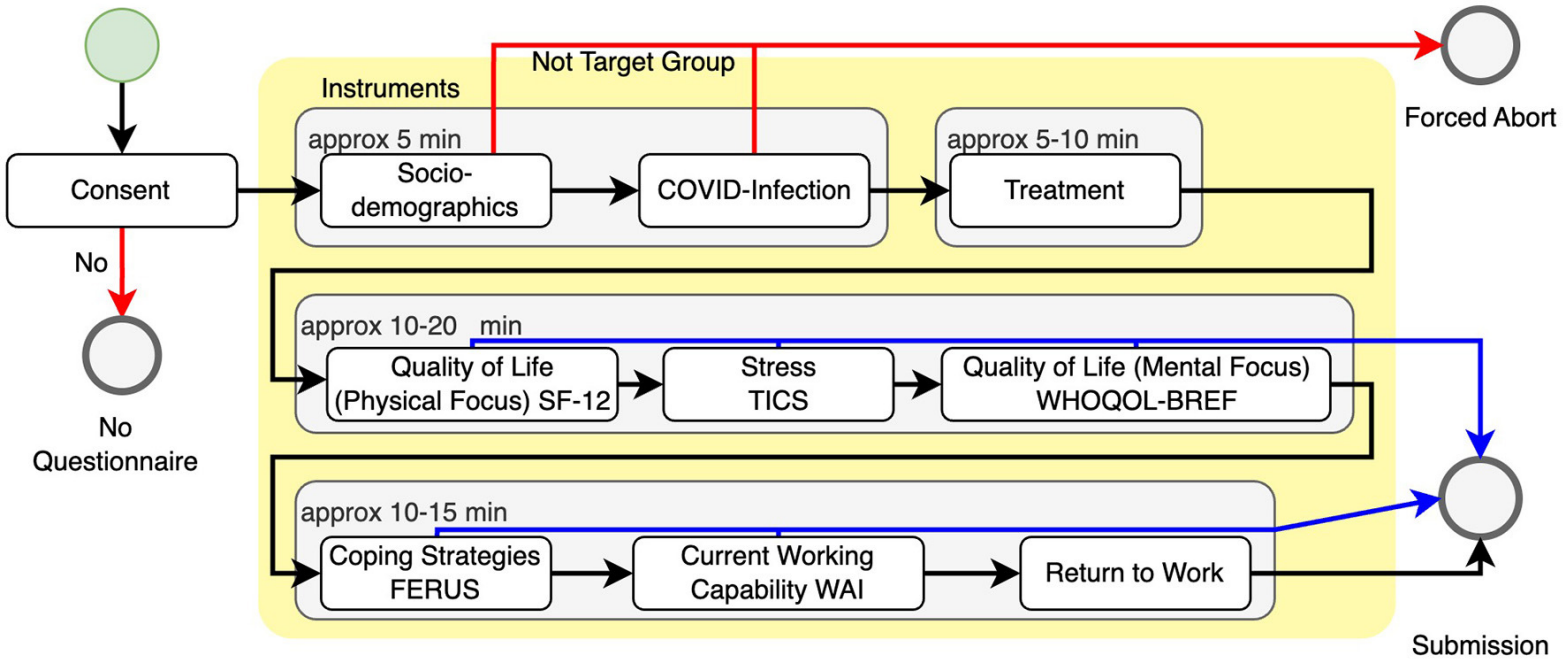
Overall study design. The user starts at the top left (green circle) and should follow the black arrows to the bottom left (gray submission circle). If they do not consent to the use of their data or are not part of the target group, the study is aborted (red arrows). After the initial questions, the user may be able to skip several instruments and complete the study early (blue arrows).

Figure 1 illustrates the procedure of the study. The black arrows indicate the envisioned sequence of questions if the participant is part of the target group and answers all questions. The questionnaire was online available from 1^*st*^ November 2022 till 31^*st*^ January 2023 and resulted in 222 data sets. Of those data sets, 184 were part of the target group and used for further evaluation.

Ethical aspects of the research, including the selection of materials and methods, were reviewed and approved by the Joint Ethics Committee of the Universities of Applied Sciences of Bavaria (GEHBa) in accordance with current scientific best-practice guidelines under vote GEHBa-202209-V-074. All participants gave their informed consent.

### 2.3 Data analysis

Data was analyzed using SPSS version 29.0 (SPSS Inc., Chicago, IL, United States). A descriptive analysis was conducted to outline personal resources, care pathways, and organizational offerings of Post-COVID affected. Mean and standard deviation were compared to describe relationships between specific results and return to work. Multiple submissions were controlled by checking internal consistency as well as dates and times of answers. To interpret scores such as the TICS score, the FERUS score, or the WAI, the average scores of the sample were compared to normative controls.

## 3 Results

Based on the cross-sectional evaluation of the 184 data sets, the majority of respondents (57%) were between 30 and 49 years old, followed by 36% of the participants being between 50 and 64 years, while the remaining 7% were either between 18 and 29 or above 64 years old. Female respondents outnumbered male participants by a total of 77%. Around 80% resided permanently in Germany, with the remaining 20% being residents in Austria. In total 58% of all respondents had worked full-time before their COVID-19 infection, 40% were employed on a part-time contract, and 2% were undergoing vocational training. In accordance with the age profile of the respondents, 83% had more than 9 years of working experience, 14% of all participants had been working between 3 and 9 years, and 3% had only 1–2 years of working experience (a minimum of 12 months working experience was an inclusion criterion). Almost half of the sample (48%) was infected with COVID-19 between 6–12 months prior to completing this survey, while 27% reported an infection between 12 and 24 months before participating in this study, and 17% had been infected more than two years ago. Only 8% were infected between 3 to 6 months prior to their participation in this study. While 90% had received a Post-COVID diagnosis from a physician, the remaining 10% suffered from symptoms other physical or mental reasons cannot explain and thus are most likely attributed to the Post-COVID syndrome. Nearly 94% of all respondents still suffered from different symptoms, and only 6% stated they had no more complaints. Eventually, 98 participants (54%) had returned to work since their COVID-19 infection, with 36% being back on their regular working hours and 18% still on reduced working hours. The remaining 82 participants indicated they had not returned to their workplace. This allowed to split the sample into the following two cohorts: (1) RTW (n_*RTW*_ = 98): participants that had returned to work after their initial infection and (2) NRTW (n_*NRTW*_ = 82) participants that had not returned to work since their COVID-19 infection.

Analysis of the GBB24 showed that being easily exhausted was the most prevalent as well as severest symptom persisting even after 3 months of the initial infection with COVID-19. A two-sample t-test indicated that the RTW cohort showed significantly (t = –4.695, *p* = 0.003) lower levels of being easily exhausted with a mean score of 2.95 (SD = 1.06) (on a 5-point Likert scale ranging from 0(=not at all) to 4(=very much) than the NRTW cohort with a mean score of 3.57 (SD =0.70). Table 1 presents mean scores for all 24 complaints assessed by the scale.

**Table 1:** Perceptions of level of complaints (GBB24): means and standard deviations.

**Complaint**	**N**	**Mean^*a, b*^**	**SD**
Being easily exhausted	182	3.23	0.964
Tiredness	182	2.77	1.146
Feeling of weakness	182	2.74	1.065
Excessive need for sleep	180	2.48	1.217
Faintness	183	2.46	1.083
Headache	182	2.24	1.259
Feeling of heaviness in the legs	181	2.22	1.385
Melalgia	181	2.07	1.417
Daze feeling	178	1.95	1.254
Feeling of pressure in the head	181	1.84	1.279
Palpitations or heart pounding	180	1.77	1.295
Dizziness	181	1.67	1.188
Shortage of breath	183	1.64	1.359
Neck or shoulder pain	180	1.42	1.337
Stabbing chest pain	182	1.25	1.313
Backache	177	1.20	1.293
Cardiac pain	180	1.15	1.301
Feeling bloated or distended	179	1.07	1.270
Lumb in the throat	178	0.72	1.120
Stomachache	179	0.58	0.964
Nausea	179	0.52	0.968
Heartburn	179	0.48	0.932
Burping	178	0.39	0.824
Vomiting	179	0.10	0.398

a measured via 5-point Likert scale with the following response options 0 “not at all,” 1 “slightly,” 2 “somewhat,” 3 “considerably,” and 4 “very much.”

b higher scores present higher level of complaints.

When assessing the different types of care or support the participants had received, nearly all respondents had consulted a general practitioner (96%). In total 62% of all participants had searched for information on the Post-COVID syndrome online, and another 56% had joined an online support group. Considerably fewer participants had been referred to a specialized Post-COVID health facility such as an inpatient rehab facility (41%), a Post-COVID ambulance (31%), or an outpatient rehab facility (6%). When exploring the reasons why less than half of the participants had received specialized Post-COVID healthcare, the answers provided a clear picture: lack of availability, as well as lack of specific information about inpatient rehab facilities (39.6%), or ambulances with specialized Post-COVID treatments (24.5%), were the most common answers given.

Being asked to rate on a 4-point Likert scale ranging from 0(=not helpful at all) to 3(=very helpful) how helpful the received treatment or care was perceived by the respondents, the mean index showed that only onsite support groups (mean index 2.45, SD = 0.69) or online support groups (mean index 2.28, SD = 0.72) were perceived as helpful or very helpful on average. In contrast, treatment at outpatient (mean 0.73, SD = 0.47) or inpatient (mean 1.75, SD = 1.021) rehab facilities as well as treatment at Post-COVID clinics (mean 1.61, SD = 0.78) and consultation of general practitioners (mean 1.24, SD = 0.84) were rated as less or not helpful at all throughout the whole sample.

To evaluate the participants' health-related quality of life, the SF-12 was applied. Results showed a meager mean index score of 12.6 (SD = 5.57) compared to the normative control range (22–28) of the validated scale. The overall score on satisfaction with the quality of life of the WHOQL-BREF reported similar results: with a mean score of 27.78 (SD = 15.81) for the NRTW cohort and of 42.84 (SD = 19.60) for the RTW cohort, results are far below normative controls of the general population (mean = 71.83, SD = 18.52). When evaluating the response scores on the different domains of the WHOQL-BREF, this relatively low score seems to be mainly affected by physical issues of the Post-COVID infected persons (mean score RTW cohort 54.99/SD = 20.81; mean score NRTW cohort 34.23/SD = 14.48; mean score normative control cohort 74.02/SD = 15.68). A two-way analysis of variance clearly denoted a significantly lower index in this domain for the NRTW cohort compared to those who had already returned to work (F = 9.267, *p* = 0.003), but not for the other domains, i.e., social interactions, environment, and psychological. Table 2 shows all means and standard deviations of the two cohorts compared to the normative control levels.

**Table 2:** Quality of life (WHOQOL-BREF): means and standard deviations of t-scores compared to the normative control group.

	**N**	**Min**	**Max**	**Mean NRTW** **(SD)**	**Mean RTW** **(SD)**	**Mean Normative** **Control (SD)**
Overall	179	.00	75.00	27.78 (15.81)	42.84 (19.60)	67.59 (17.93)
Domain environment	177	21.88	100.00	66.09 (15.29)	74.05 (13.92)	70.38 (14.17)
Domain physical	174	7.14	96.43	34.23 (14.48)	54.99 (20.80)	76.92 (17.68)
Domain psychological	178	10.71	100.00	51.67 (17.17)	60.33 (17.57)	74.02 (15.68)
Domain social relationships	173	.00	100.00	62.87 (20.79)	64.58 (21.56)	71.83 (18.52)

Besides physical issues that affected the participants' quality of life, the study revealed stressors that might also lead to significantly lower levels of quality of life in the Post-COVID affected population. Therefore, responses to four sub-scales of the TICS questionnaire were analyzed. A comparison of mean scores showed that the RTW cohort particularly suffered from higher levels of social overload (mean score 12.22/SD = 4.99) compared to the normative controls (mean score 9.70/SD = 5.23). In contrast, the major stressor for the NRTW cohort was found to be social isolation (mean score 8.88/SD = 5.42) compared to the normative controls (mean score 6.22/SD = 4.84). Table 3 illustrates further details of the TICS results.

**Table 3:** Stressors (TICS) means and standard deviations compared to the normative control group.

	**N**	**Min**	**Max**	**Mean NRTW** **(SD)**	**Mean RTW** **(SD)**	**Mean Normative** **Control (SD)**
Social overload	163	0.00	24.00	9.18 (5.51)	12.22 (4.99)	9.70 (5.23)
Social isolation	165	0.00	24.00	8.88 (5.42)	7.13 (5.15)	6.22 (4.84)
Social tensions	166	0.00	24.00	5.41 (3.99)	5.44 (3.90)	5,69 (3.91)
Lack of social recognition	164	0.00	16.00	4.38 (3.56)	5.42 (3.55)	4.48 (3.18)

To evaluate which type of personal resources might lead to a successful return to work, the FERUS scale revealed the following: While a comparison of the mean score of the sub-scale social support did not show any significant difference between both cohorts, the sub-scale coping showed a significant difference (F = 4.913, *p* = 0.001). For participants that had returned to work, a mean score of 49.33/SD = 11.06) was calculated and found to be within the range of the normative control (40–60). However, for the NRTW cohort, a significantly lower mean score of 35.92/SD = 10.47 was calculated. Table 4 shows the means and standard deviations of the FERUS scale.

**Table 4:** Personal resources (FERUS)—means and standard deviations of t-scores compared to the normative range.

	**N**	**Min**	**Max**	**Mean NRTW** **(SD)**	**Mean RTW** **(SD)**	**Mean Normative** **Control (SD)**
Coping	174	26.00	74.00	35.92 (10.47)	49.33 (11.06)	40–60
Social support	165	26.00	44.00	37.98 (5.00)	35.96 (5.41)	40–60

The work ability index (WAI) of the total sample was 24.9, which is referred to as poor work ability on a scale ranging from 7 to 49. Only index scores from 37 onwards denote good work ability. On average, participants returned to work after 9.45 weeks (SD = 13.95) after their initial COVID-19 infection, with a maximum of 78 weeks after initial infection with the virus. In terms of organizational provisions and offerings for reintegration into work-life, results showed a heterogeneous picture. Nearly half of the cohort (*n* = 42) were allowed to reduce working hours and/or were offered an occupational reintegration plan (*n* = 39). Moreover, structural changes such as adjusting the workplace (*n* = 35) or tasks (*n* = 29) were offered to Post-COVID affected. Health courses (*n* = 28), as well as general consultation (*n* = 31) and job coaching (*n* = 12), were also provided. However, when being asked to rate on a scale from 0 (=not helpful) to 3 (=very helpful) how helpful the offerings were perceived in order to facilitate successful reintegration into work-life, only structural changes such as modification of the workplace, working hours and task were rated as helpful or very helpful on average (mean_*workplace*_ 2.55/SD = 0.83, mean_*workinghours*_ 2.44/SD = 0.80; mean_*tasks*_ 2.55/SD = 0.83), while the remaining offerings were rated as less helpful or not helpful at all.

## 4 Discussion

The conducted study performed an in-depth evaluation of care pathways, personal resources, and organizational offerings that might facilitate the individuals' return to work after or while still being affected by the Post-COVID syndrome. As a novelty, this study has taken a Post-COVID patients centric approach to understand what may be crucial for a successful transition from care pathways back to corporate life.

First, results showed that the overall and health-related quality of life of the sample was significantly diminished compared to normative controls. While here it it can be argumented that a decline in health-related quality of life amid the pandemic might not only be related to the Post-COVID syndrome but can also be attributed to specific workplace settings and procedures such as wearing personal protective equipment that led in some cases to e.g., dermatological issues (31, 32), the study was able to provide further insights. With a relatively low average work ability index of 24.9 across the whole sample, Post-COVID patients seem to be strongly affected in their work ability. Hence, it can be assumed that e.g., external workplace seetings might be not fully able to explain reduced quality of life. These observations correlate with the findings of a recent study that also demonstrated a substantial impact of the Post-COVID syndrome on the work ability of an occupational cohort (5) and stresses at the same time the relevance of this study to research on the subject of Post-COVID and its impact, particularly on the working population. In this context, the study was able to provide further insight into reasons for such high impacts on the Post-COVID patients' work ability. As shown, being easily exhausted was the most prevalent symptom, followed by tiredness, feeling of weakness, excessive need for sleep, and faintness – all complaints that can be summarized under the phenomena of fatigue. More detailed analyses showed that the sub-cohort NRTW showed significantly higher levels of these fatigue symptoms than those participants that had already returned to work. Therefore, in accordance with earlier research in the realm of chronic fatigue and Post-COVID (33–35), these findings can be considered preliminary evidence that fatigue and exhaustion are essential predictors of an individual's level of work ability.

In light of the above, the results were able to demonstrate another critical aspect: The majority of participants, that completed rehabilitation programs or received treatments or consulting at Post-COVID ambulances, rated those offerings as not or little helpful with regard to their way back into working life. On the contrary, the entire sample rated both inpatient and outpatient rehab facilities as well as Post-COVID outpatient clinics as less helpful or not helpful in the return to work process. Analysis of the responses to the open questions provided further explanation of these findings, with additional comments stating that the received treatments even worsened the level of exhaustion and fatigue, resulting in extended rest and recovery needs incompatible with work demands. These findings lead to the conclusion that at an early stage of a Post-COVID diagnosis, all patients need to be administered a validated measure of fatigue such as the Fatigue Severity Scale (FSS), the Multi-Dimensional Assessment of Fatigue (MAF), or the Multi-Dimensional Fatigue Inventory (MFI) (36) to predict the individuals' work ability at an early stage of treatment. Following a recent scholarly discussion (37), this results in the practical implication that fatigue management strategies have to be included in all types of care pathways, given their high relevance for the individuals' quality of life but also occupational health.

Then, the findings provided enlightening results regarding personal resources that might facilitate the return to work after being affected by the Post-COVID syndrome. While both subgroups (RTW and NRTW) showed similar results in terms of social support, there was a significant difference in the participants' ability to cope with stressors. Those who had already returned to their workplaces showed an average score of 49.33 (SD = 11.06) for their coping levels, comparable to the normative controls on the applied FERUS scale. However, those participants who had not returned to work so far showed considerably lower levels of coping abilities. These results suggest that the ability to cope with health stressors might be another vital determinant when it comes to the individuals' journey back into work-life. While there exists earlier literature in the area of coping, resilience, and health-related stressors such as breast cancer (38), HIV (39), or heart diseases (40), that back those findings of the present study, there is still a lack of in-depth research on coping and the Post-COVID syndrome. However, existing evidence from those studies might be transferred and applied to Post-COVID treatment plans. By integrating the patients' education on psychosocial coping techniques widely into all types of care pathways and offerings, the patients' quality of life and successful return to occupational life might be fostered.

Finally, when it comes to organizational offerings supporting Post-COVID patients' return to work, the study reinforced that structural measures such as reduced working hours, level of tasks, and working environment were perceived as most helpful from the employees' perspective. These observations contrast with earlier findings of studies with occupational cohorts that suffered from, e.g., heart or musculoskeletal diseases, where workplace health management measures produced positive outcomes (41). These contrasts can be attributed to the fact that the cohort mainly suffered from fatigue as a cardinal symptom, so sports and physical activity might not be suitable to improve their condition. As was shown in earlier studies in the context of chronic fatigue and physical activities (35). Moreover, as the analysis of the open responses on the effectiveness of organizational offerings showed, participants also emphasized the importance of open communication with employers, colleagues, and healthcare providers to facilitate understanding and accommodate their unique needs during the transition back to work. These findings lead to the conclusion that organizational offerings for Post-COVID patients have to include modifications to work environments, schedules, and tasks but also have to cater to their specific needs, such as education on how to pace one selves' resources to match energy levels with work activities, prioritize rest and cope with demanding work environments. Those needs imply that existing workplace reintegration must be revived and revised according to the unique needs of Post-COVID affected employees. In this context, digital tools might be an innovative approach, as previous research has shown that, particularly in areas where multidisciplinary care is needed, e-approaches could support patients at the interface between medical care, e.g., inpatient rehab treatments, and their return to work (42–44).

This study presented has some limitations. One major limitation concerns the sample size. We calculated a minimum sample size of *n* = 385, assuming the prevalence of Post-COVID within the population was between 5 and 10% and a confidence interval of 95%. After talking to experts from involved Post-COVID clinics and rehab facilities, 200 participants. They assumed that a large proportion of Post-COVID patients are still not diagnosed or have not consulted a Post-COVID ambulance, rehab facility, or joined a support group, so they might not be reached by this study's recruitment strategy. As described in Section 2.2, 222 data sets were collected, whereas 184 met the inclusion criteria and were analyzed further. In this light, the presented study overachieved the expectations of experts. As this study provides some in-depth description of patients' needs and paves the way for further investigations, statistical significance was not the main focus. Although the recruitment strategy was well designed, reaching out to post-COVID patients proved quite tricky. This may be explained by the fact that those who had already returned to work were too busy with their daily tasks, so they could not join the study. Then, as mentioned before, those still at home suffered from high levels of fatigue, which led to the fact that some of them struggled to respond to a questionnaire with 148 items in one sitting.

Another limitation of this study was the composition of the cohort. Although the recruitment strategy was intended to produce a heterogenous sample, female respondents outnumbered male respondents by far. Furthermore, the age groups between 18 and 30 and above 64 were underrepresented. While the latter probably derives from the fact that the recruitment channels did not reach those age groups as well as inclusion criteria excluded retirees, the former might be attributed to earlier studies' findings, which stressed that women are more prone to suffer from Post-COVID syndrome than men (19). Although these limitations might challenge the generalizability of the results, the findings foster a comprehensive understanding and indications of the perspective and needs of Post-COVID patients pertaining to their return to work. Nevertheless, future research in Post-COVID and return to work should consider shorter questionnaires to produce larger sample sizes and prevent a high dropout rate. However, as this study has shown, it seems worthwhile to investigate further topics such as personal resources and coping strategies of Post-COVID affected to learn more about different patterns of coping strategies applied, such as the patients' ability to seek or use social support, behavioral escape-avoidance, or focusing on the positive. Here, it may be instructive to use qualitative study designs to produce a more profound understanding from the patient's perceptive.

## Data availability statement

The raw data supporting the conclusions of this article will be made available by the authors, without undue reservation.

## Ethics statement

The studies involving humans were approved by Gemeinsame Ethikkommission der Hochschulen Bayerns Hochschule Bayern e.V., Hohenzollernstraße 102, 80796 München. The studies were conducted in accordance with the local legislation and institutional requirements. The participants provided their written informed consent to participate in this study.

## Author contributions

CS: Conceptualization, Formal analysis, Funding acquisition, Investigation, Methodology, Resources, Validation, Visualization, Writing—original draft, Writing—review & editing. DH: Data curation, Formal analysis, Investigation, Resources, Software, Visualization, Writing—original draft, Writing—review & editing. MK: Conceptualization, Funding acquisition, Project administration, Resources, Writing—review & editing. MJ: Conceptualization, Funding acquisition, Methodology, Project administration, Resources, Supervision, Writing—review & editing. JS: Conceptualization, Funding acquisition, Project administration, Resources, Software, Supervision, Writing—review & editing.
